# Metabolite-mediated mechanisms linking the urinary microbiome to bladder cancer

**DOI:** 10.71150/jm.2509001

**Published:** 2025-11-30

**Authors:** Thu Anh Trần, Ho Young Lee, Hae Woong Choi

**Affiliations:** Division of Life Sciences, Korea University, Seoul 02841, Republic of Korea

**Keywords:** bladder cancer, urinary microbiome, microbial metabolites, tumor microenvironment

## Abstract

Bladder cancer is the most common malignancy of the urinary tract and is a major health burden globally. Recent advances in microbiome research have revealed that the urinary tract harbors a resident microbial community, overturning the long-held belief in its sterility. Increasing evidence suggests that microbial dysbiosis and microbially derived metabolites contribute to bladder cancer carcinogenesis, progression, and therapeutic responses. Distinct microbial signatures have been observed in bladder cancer patients, with notable differences across disease stages and between primary and recurrent cases. Mechanistic studies have demonstrated that microbe-associated metabolites and toxins can drive DNA damage, chronic inflammation, extracellular matrix remodeling, and epithelial–mesenchymal transition. In addition, biofilm formation allows bacteria to evade immune responses and promotes persistent inflammation, creating a tumor-permissive niche. Beyond pathogenesis, microbial activity also influences therapeutic outcomes; for instance, some microbial pathways can inactivate frontline chemotherapy, while others generate metabolites with anti-tumor properties. Collectively, these patterns define a microbiota–metabolite–immunity axis, presenting opportunities for precision oncology. Targeting microbial pathways, profiling urinary microbiota, and harnessing beneficial metabolites offer promising advancements in biomarker discovery, prognostic refinement, and the development of novel therapeutic strategies for bladder cancer.

## Introduction

Bladder cancer represents the most common malignancy of the urinary tract. According to global cancer statistics from 2022, more than 613,800 new cases and 220,300 deaths were reported worldwide that year (Bray et al., 2024). Based on the depth of invasion into the bladder wall, bladder cancer is divided into two major categories: non–muscle invasive bladder cancer (NMIBC), which comprises about 75% of cases, and muscle–invasive bladder cancer (MIBC), which accounts for the remaining 25% (Cumberbatch et al., 2018). NMIBC is clinically significant for its remarkable tendency to recur; within five years, 50–70% of patients will experience recurrence, and a subset of these recurring cases will progress to MIBC (Al Awamlh and Chang, 2023; Tan et al., 2016). In contrast, MIBC is notable for its aggressiveness; it carries a high potential for metastasis, and the five–year survival rate falls precipitously—from nearly 80–90% in localized cases to less than 10% in metastatic cases (Ripoll et al., 2021; Wang et al., 2018). Age represents a strong determinant of incidence, with bladder cancer occurring most frequently in older adults. Sex also modifies risk, as men develop bladder cancer at nearly three times the rate observed in women. Finally, among environmental risk factors, cigarette smoking and exposure to occupational or industrial carcinogens remain the best–established contributors to disease burden.

In recent years, the role of the human microbiome in both the maintenance of health and the development of disease has gained increasing attention. A growing body of evidence suggests that microorganisms can act as central players in cancer biology, with nearly 20% of all cancers estimated to be associated with specific microbial factors (de Martel et al., 2012). Several well–established examples illustrate this principle: *Helicobacter pylori* serves as a major etiologic agent of gastric cancer, *Fusobacterium nucleatum* promotes tumor progression in colorectal cancer, and dysbiosis of the host microbiota has been implicated in breast cancer pathogenesis (Amieva and Peek, 2016; Parida and Sharma, 2019; Wang and Fang, 2023). Especially, the colonization of *Fusobacterium nucleatum* reduced intratumoral T cell infiltration, creating an immunosuppressive environment favorable to cancer progression (Parhi et al., 2020). Such microbial dysbiosis perturb host homeostasis, creating conditions that can drive carcinogenesis. In light of these precedents, the possibility that the urinary microbiome (urobiome) may contribute to bladder cancer development is a necessary consideration.

For much of the past century, the bladder epithelium and urine were regarded as sterile environments. Recent studies, however, have revealed that the urinary tract harbors a distinct microbiome. The detection of microbes in urine does not necessarily signify infection. While some species indeed provoke acute infection or chronic inflammation, others exert protective effects by suppressing the growth of pathogenic bacteria. These host–microbe interactions are highly complex and can influence carcinogenesis, tumor progression, and even therapeutic responses through a diverse range of molecular mechanisms. For example, microorganisms with distinct biochemical activities can intervene in the metabolism and excretion of host carcinogens, amplifying or attenuating their harmful potential. In one such case, nitrate–reducing bacteria have been shown to enhance the generation of carcinogenic N-nitrosamines (Carlström et al., 2020; Lundberg and Weitzberg, 2013). In addition, toxic compounds, such as heavy metals, pesticides, ochratoxin, and polycyclic aromatic hydrocarbons (PAHs), are filtered from the blood by the kidneys and concentrated in the bladder, where they interact with the resident urinary microbiota. The metabolites arising from this interplay may either heighten or diminish the risk of bladder cancer development.

This review examines the characteristics of the urobiome in patients with bladder cancer and compares them with those of healthy controls, exploring the possibility that microbial communities and their metabolites contribute to bladder carcinogenesis. We suggest that specific microbial signatures could serve as diagnostic or prognostic biomarkers for bladder cancer. Moreover, the research to date raises the prospect that modulation of the microbiome may form the basis for adjunctive therapeutic strategies that could be implemented alongside conventional anticancer treatments.

## Urobiomes in Healthy Subjects

Advances in sampling and analytic technologies have dramatically expanded our understanding of the urobiome. These developments provide the foundation for investigating the roles of resident microbiota in urinary tract health and disease pathophysiology. Compared with the gastrointestinal tract and other sites in the body, the urobiome is characterized by lower overall microbial abundance and diversity. At the phylum level, men and women show remarkably similar urobiome profiles. *Firmicutes* predominate, followed by *Actinobacteria*, *Bacteroidetes*, and *Proteobacteria*, and these four phyla together account for more than 97% of taxa identified in urine and bladder tissue (Mansour et al., 2020; Modena et al., 2017). At the genus level, more striking differences emerge. While *Prevotella*, *Escherichia*, *Enterococcus*, *Streptococcus*, and *Citrobacter* are commonly found in both sexes, certain genera display sex specificity (Modena et al., 2017). *Pseudomonas*, for example, is highly reported in men, and both *Corynebacterium* and *Streptococcus* appear more abundant in male urine (Fouts et al., 2012; Modena et al., 2017). In contrast, *Lactobacillus* dominates the female urobiome and is widely regarded as a hallmark of urinary health in women (Fouts et al., 2012; Song et al., 2022).

Age introduces a layer of variability that transcends sex. Across older populations, regardless of sex, the overall abundance of urinary microbes generally declines, being influenced by hormonal changes, diet, hygiene, and physiological aging (Colella et al., 2023). Intriguingly, despite a decline in bacterial load, one study reported greater microbial diversity in individuals over 70 years of age, identifying four genera—*Jonquetella*, *Parvimonas*, *Proteiniphilum*, and *Saccharofermentans*—that appeared exclusively in this age group (Lewis et al., 2013). Taken together, these findings demonstrate that the urobiome is far from static. Rather, it is dynamically shaped by sex, age, hormones, and lifestyle, highlighting the need for personalized approaches in evaluating its role in bladder health and carcinogenesis.

## Methodological Influences on Bladder Cancer Urobiome Studies

Most studies on the urobiomes of bladder cancer patients have compared microbial features between patients and healthy individuals, focusing on alterations in urinary diversity and composition that might relate to cancer initiation, progression, or treatment responses. Results from these studies, however, are not always consistent. Differences in sample collection methods, patient demographics, and disease stage have led to conflicting interpretations, underscoring the need for refined analyses and standardized research designs. Urine samples from bladder cancer patients can be obtained in several ways, including urinary catheterization and bladder washout fluid and midstream urine collection. Catheterized urine tends to display lower genus-level diversity than midstream urine samples, yet catheterization minimizes contamination from the external environment and is therefore often regarded as a more reliable collection method for urobiome studies (Bukavina et al., 2023). Importantly, microbial profiles vary significantly according to collection method (Oresta et al., 2021). In catheterized urine, bladder cancer patients frequently exhibit higher levels of *Veillonella* and *Corynebacterium* than healthy individuals, while *Ruminococcus* is reduced. In bladder washout samples, *Burkholderiaceae* is preferentially enriched, suggesting the localization of certain taxa within the bladder lumen. By contrast, midstream urine frequently exhibits increased abundances of *Streptococcus*, *Enterococcus*, *Corynebacterium*, and *Fusobacterium*, reflecting contributions from both Gram-positive and anaerobic species. Overall, research has made it clear that sampling methodology exerts a decisive influence on urobiome composition and that standardization is essential for identifying bladder cancer–specific microbial signals. Beyond methodological considerations, recent functional evidence has also underscored the pathological importance of dysbiosis: urinary microbiome alterations have been shown to associate with inflammation and disrupted fatty acid metabolism, thereby linking dysbiosis directly to bladder cancer pathogenesis (Wu et al., 2024).

Tissue-based analyses provide an additional perspective. In one study, tumor specimens were collected during transurethral resection of bladder tumor (TURBT), while urine samples were obtained concurrently from the same patients using resectoscopy (Mansour et al., 2020). Microbiome profiling across multiple regions of the same tumor revealed highly consistent community structures, indicating that tissue-based signatures are spatially stable within the tumor. When the tissue and urine samples from the same individual were compared, several genera—including *Akkermansia*, *Bacteroides*, *Clostridium* sensu stricto, *Enterobacter*, and *Klebsiella*—were significantly more abundant in tissue. These five taxa have been proposed as “suspect genera” indicative of bladder cancer. Additionally, *Ralstonia* has emerged as a particularly notable taxon: it has repeatedly been detected in bladder cancer tissue, with species such as *Ralstonia* sp000620465, *R. pickettii*, and *R. mannitolilytica* found to be especially enriched in muscle-invasive bladder cancer (MIBC) (Liu et al., 2019; Sun et al., 2023). Such findings indicate that tissue-based analyses may more faithfully capture tumor microenvironment–specific microbial signatures than urine-based analyses.

Although heterogeneity in study designs, different sampling approaches, and analytic sensitivity have prevented a complete consensus, the accumulating evidence points in the same direction. Dysbiosis of the urobiome—manifesting through the enrichment or depletion of particular taxa—appears to be linked to bladder cancer. Clarifying the pathological significance of these associations will require standardized methodologies, larger patient cohorts, and functional analyses capable of distinguishing causal roles from mere correlation.

## Urobiome Diversity in Bladder Cancer Patients

A substantial body of research exists investigating the relationship between the urobiome and bladder cancer. Across multiple studies, urine from bladder cancer patients has shown significantly increased microbial diversity and richness, with β-diversity analyses consistently demonstrating statistically significant differences between patients and healthy controls (Hussein et al., 2021; Sheng et al., 2025a). Other studies reporting elevated α-diversity in bladder cancer patients have interpreted this finding as greater microbial evenness (Oresta et al., 2021). In a recent analysis based on midstream urine samples, bladder cancer patients exhibited significantly higher Shannon index values than healthy individuals, and this increase in diversity was proposed to reflect a history of recurrent urinary tract infections (Sheng et al., 2025a). Paradoxically, another investigation found that lower α-diversity—particularly reduced Shannon indices—correlated with prolonged recurrence-free survival (Zeng et al., 2020). Taken together, these observations suggest that bladder cancer’s effects on diversity per se cannot be understood simply as “higher” or “lower.” Rather, biological meaning likely depends on the composition of the community, the host’s clinical history (e.g., urinary tract infections), and interactions within the tumor microenvironment. Indeed, early studies that overlooked such factors are now recognized to have contributed to the inconsistencies in results.

Demonstrating geographic variation, *Firmicutes* has repeatedly been reported as the most abundant phylum in both the urine and tissue samples of bladder cancer patients (Bučević Popović et al., 2018), but several China-based studies identified *Proteobacteria* as the dominant taxon in both sample types (Liu et al., 2019; Wu et al., 2018). Additionally, gender-specificity in the urobiome appears to persist even among bladder cancer patients. Bladder cancer-positive women demonstrate relatively higher proportions of *Bacteroidetes*, while bladder cancer-positive men show greater abundances of *Actinobacteria*. At the genus level, *Pelomonas*, *Corynebacterium*, and *Finegoldia* are enriched in men, whereas *Lactobacillus*, *Actinotignum*, and *Prevotella* predominate in women (Hussein et al., 2021; Kustrimovic et al., 2024). Such findings indicate that sex-based microbial differences remain evident under disease conditions, suggesting that sex may represent an important variable for future diagnostic and therapeutic strategies.

**Fig. 1.**
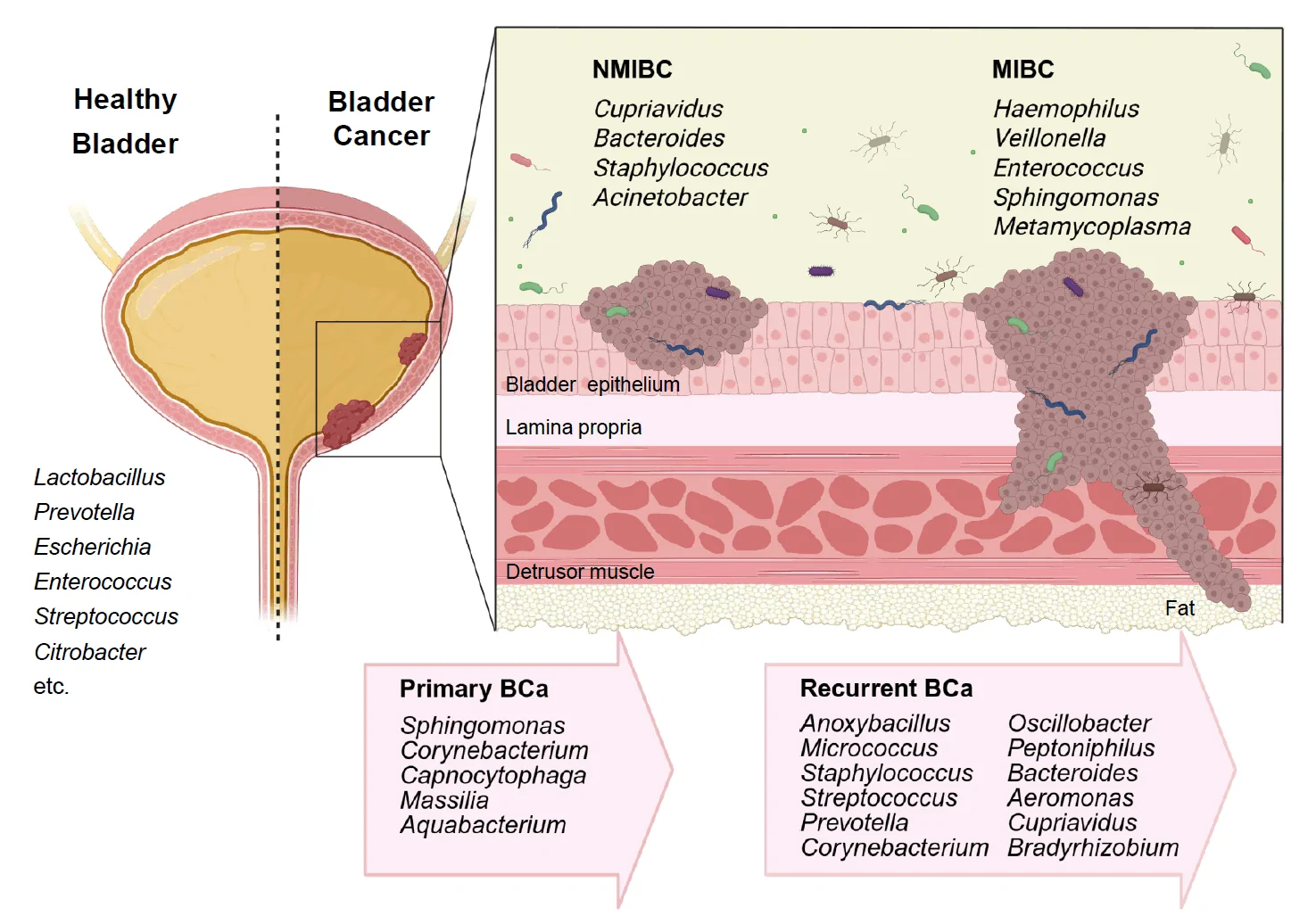
Distinct microbiome profiles across healthy, non–muscle invasive, and muscle-invasive bladder cancer. Comparison of microbial composition in healthy bladder versus bladder cancer highlights distinct enrichment of specific genera. Differences are observed between non–muscle invasive bladder cancer (NMIBC) and muscle-invasive bladder cancer (MIBC), as well as between primary and recurrent cases (BCa).

Overall, these studies converge on the conclusion that the diversity and composition of the urobiome in bladder cancer patients are shaped not only by disease status but also by clinical and demographic factors, such as diagnosis timing, disease stage, and patient sex. These patterns are summarized in Fig. 1, which illustrates microbial alterations associated with bladder cancer across healthy controls, primary and recurrent cases, and non–muscle invasive versus muscle-invasive disease. Building on this foundation, the following section examines how microbial communities differ between newly diagnosed and recurrent bladder cancer, as well as across distinct tumor stages.

### Distinct microbiota associated with recurrent and primary bladder cancer

Recurrent bladder cancer patients differ fundamentally from those newly diagnosed, as they have typically undergone prolonged courses of anticancer therapy. Such prior treatments introduce an additional variable, making microbiome differences between patients and healthy individuals largely expected. In contrast, as patients with primary bladder cancer have not yet received such therapy, their microbiota offer a clearer window into potential causal relationships between microbial dysbiosis and bladder cancer development. A study of 170 bladder cancer patients provides support for this distinction. Among participants, 125 were newly diagnosed with primary bladder cancer (BCa_P), while 45 had recurrent disease (BCa_R) (Sheng et al., 2025b). Measures of α-diversity were significantly higher in BCa_P patients than in BCa_R patients, and β-diversity analyses revealed distinct microbial community structures in the two groups. Taxonomic differences were also evident: *Sphingomonas*, *Corynebacterium*, *Capnocytophaga*, *Massilia*, and *Aquabacterium* were enriched in BCa_P urine, whereas *Aeromonas*, *Cupriavidus*, and *Bradyrhizobium* predominated in BCa_R samples. Additional studies have reinforced these findings, and genera like *Micrococcus*, *Brachybacterium*, and *Acinetobacter* have been added to those associated with recurrence or progression (Zeng et al., 2020; Zhang et al., 2023). Patients at high risk of recurrence or progression have also demonstrated increased abundances of *Herbaspirillum*, *Porphyrobacter*, and *Bacteroides*, suggesting that enrichment in certain microbial taxa could serve as biomarkers of disease aggressiveness (Wu et al., 2018). Functional prediction analyses can serve to further distinguish patient groups. In BCa_P patients, microbial pollutant degradation pathways and microbial tricarboxylic acid cycle were preferentially activated, while in BCa_R patients, microbial pathways related to glucose metabolism and oxidative stress were upregulated.

Thus, recurrent bladder cancer appears to be characterized by reduced microbial diversity and the selective expansion of potentially pathogenic genera. Such consistent differences in microbial composition and metabolic pathway activation between newly diagnosed and recurrent bladder cancer patients underscore a central conclusion: urobiome dysbiosis may contribute both directly and indirectly to bladder cancer initiation and its subsequent progression.

### Stage-specific microbiome shifts in bladder cancer

Multiple studies have consistently demonstrated that the microbial communities associated with NMIBC differ markedly from those associated with MIBC. As tumor stage advances, microbial communities undergo significant change, and β-diversity, which captures differences between communities, increases (Wu et al., 2018). These shifts often include the enrichment of particular genera that correlate with tumor progression, tissue invasion, and increased risk of recurrence.

In NMIBC, several dominant genera have repeatedly been identified, including *Acinetobacter*, *Anoxybacillus*, *Brevibacillus*, *Cupriavidus*, *Staphylococcus*, *Lactobacillus*, *Bacteroides*, and *Sphingobium* (Boban et al., 2025; Hussein et al., 2021; Li et al., 2025; Sun et al., 2023). Among these, *Lactobacillus*—a member of the *Firmicutes* phylum—has drawn special attention because its relative abundance is associated with favorable responses to *Bacillus Calmette–Guérin* therapy and with lower recurrence risk (Boban et al., 2025). This observation suggests that beneficial taxa may exert protective effects in early stages of the disease.

On the other hand, in MIBC, overall microbial diversity tends to decline, while clusters of pathogenic genera become more prominent. Urine from MIBC patients has shown the relative enrichment of *Acinetobacter*, *Bacteroides*, *Chryseobacterium*, *Faecalibacterium*, *Oscillatoria*, *Ralstonia*, *Haemophilus*, *Peptoniphilus*, *Sphingomonas*, and *Veillonella* (Hussein et al., 2021; Li et al., 2021, 2025; Sheng et al., 2025a; Sun et al., 2023). These compositional changes reflect not only a loss of protective mutualists but also the emergence of taxa more directly implicated in tumor aggressiveness. Of particular mechanistic relevance is an association between the microbiome and epithelial–mesenchymal transition (EMT), a key driver of invasion in MIBC. The expression of EMT-related genes—including E-cadherin, vimentin, *SNAI2/3*, and *TWIST1*—correlates strongly with the abundances of *Escherichia coli*, *Oscillatoria*, and butyrate-producing bacterium SM4/1 (Li et al., 2021). Thus, specific microbes may contribute to the activation of EMT pathways, thereby enhancing tumor invasiveness and metastatic potential.

Based on the findings summarized above, we organized in Table 1 how microbiota is associated with the development of bladder cancer. Clinically, these differences between NMIBC and MIBC in microbial composition hold substantial implications. In addition, distinct microbial signatures have also been observed between patients with primary and recurrent bladder cancer, further underscoring the dynamic nature of the urobiome across disease progression. They may serve as biomarkers for staging, prognosis, and therapeutic stratification, while also raising the possibility of interventions that selectively target harmful taxa or restore protective ones. In this way, the stage-specific microbiome becomes a critical lens through which to understand the evolving tumor microenvironment and the biological complexity of bladder cancer.

## Microbial Metabolites and Bladder Cancer Progression

**Fig. 2.**
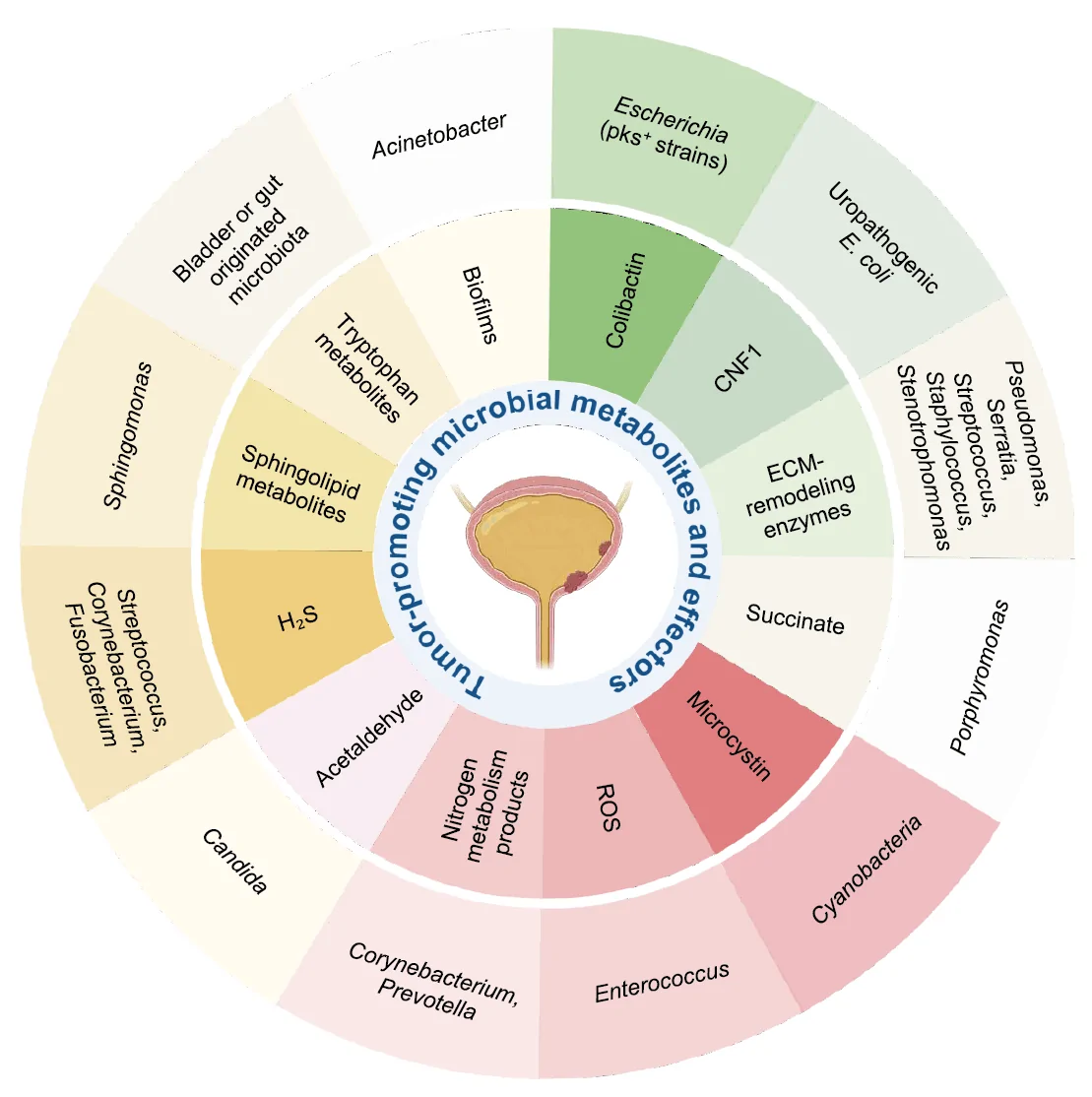
Tumor-promoting microbial metabolites and effectors in bladder cancer. Specific microbes contribute to bladder cancer development through production of metabolites such as acetaldehyde, hydrogen sulfide, sphingolipid and tryptophan derivatives, or by forming biofilms. Additional effectors—including toxins (e.g., colibactin, CNF1, and microcystin), reactive oxygen species, and ECM-remodeling enzymes—facilitate DNA damage, chronic inflammation, and tumor progression.

Although numerous studies have provided important clues linking intravesical microbial communities to bladder cancer, details of their pathological associations remain incompletely defined. Recent investigations have increasingly suggested that specific microbes—or their metabolites—contribute directly to carcinogenesis, and mechanistic insights into these processes are steadily expanding. Of particular interest are a number of microbial products, such as colibactin, cytotoxic necrotizing factor 1 (CNF1), reactive oxygen species (ROS), succinate, acetaldehyde, hydrogen sulfide, sphingolipids, and tryptophan-derived metabolites, that have been reported to inflict direct damage on bladder epithelial cells or shape the tumor microenvironment in ways that promote malignancy. In addition to metabolites, microbial behaviors like biofilm formation and extracellular matrix (ECM) degradation can perpetuate chronic inflammation and influence tissue remodeling, thereby fostering invasiveness and recurrence. These tumor-promoting microbial metabolites and effectors are summarized in Fig. 2 and Table 2, providing a conceptual framework for understanding how microbes contribute to bladder cancer development. Together, these microbe–metabolite–host interactions hold profound implications for the pathophysiology of bladder cancer and for the biological heterogeneity of tumor progression. A systematic synthesis of findings related to these interactions is now required to clarify their pathological relevance. This review, therefore, examines the principal microbial metabolites reported to affect bladder cancer and considers their pathological functions in light of the most recent evidence.

### Colibactin as a genotoxic link to bladder cancer

Although numerous bacterial species have been detected within the bladder, definitive evidence linking them directly to bladder cancer remains limited. In particular, no study has yet demonstrated beyond doubt that bacteria themselves induce the oncogenic mutations required for tumor initiation. By contrast, the gut microbiome has provided clear species–cancer links: diverse intestinal taxa have been associated with colorectal cancer development (Gagnaire et al., 2017). Among them, *Escherichia coli* strains carrying the pks pathogenicity island can produce colibactin, a genotoxin known to alkylate adenine residues and cause double-strand DNA breaks in cultured cells (Nougayrède et al., 2006). This mechanistic link was elegantly demonstrated using human intestinal organoids. Repeated luminal injections of pks^⁺^ *E. coli* over a five-month period produced a distinct mutational signature, absent in isogenic pks-mutant controls. Whole-genome sequencing confirmed that this signature was detectable in a subset of 5,876 human cancer genomes—most notably in colorectal cancer (Pleguezuelos-Manzano et al., 2020). These findings established for the first time that exposure to colibactin-producing bacteria can leave a permanent, identifiable mutational imprint.

Emerging evidence now extends this paradigm into the urinary tract. One study reported the detection of C14-Asn, a colibactin metabolite, in the urine of patients with pks^⁺^ uropathogenic *E. coli* (UPEC), providing direct evidence that colibactin is produced in the urinary tract (Chagneau et al., 2021). Furthermore, during intravesical instillation of *E. coli*, colibactin not only accumulated in the bladder but also induced DNA damage in urothelial cells, particularly in Krt14^⁺^ progenitor cells—suggesting a potential initiating event for bladder carcinogenesis (Chagneau et al., 2021). Consistent with this hypothesis, colibactin-associated mutational signatures have now been identified not only in colorectal cancer but also in urinary tract malignancies (Dziubańska-Kusibab et al., 2020).

While smoking and occupational solvent exposure remain the best-established risk factors for bladder cancer, the presence of *E. coli* has historically been overlooked (Saginala et al., 2020). A large, international case–control study, however, demonstrated that recurrent urinary tract infection is epidemiologically associated with increased bladder cancer risk (Vermeulen et al., 2015). In this context, pks^⁺^ UPEC may represent an underappreciated contributor, particularly in patients with asymptomatic, chronic infections. Indeed, C14-Asn has been detected in the urine of patients with asymptomatic bacteriuria—an often-untreated condition that can persist for years (Lindberg et al., 1975). These findings demonstrate a need for systematic approaches to define the clinical impact of colibactin production in the urinary tract. Such strategies might include the routine screening of UPEC isolates for the presence of the pks island or the direct detection of C14-Asn in urine from high-risk patients. Colibactin biosynthesis, though energetically costly, may promote long-term bacterial persistence in the urinary tract by producing both genotoxic metabolites and immunomodulatory compounds, such as C12-Asn-GABA. Notably, pks^⁺^ strains of *Klebsiella pneumoniae*—a frequent cause of catheter-associated and hospital-acquired UTIs—have also been reported in metagenomic and genomic surveys of urinary isolates since 2020.

Taken together, these observations identify colibactin as a plausible mechanistic link between *E. coli* and bladder cancer. What is urgently required now are rigorous studies to determine whether this genotoxin functions as a direct driver of human bladder carcinogenesis and whether its mutational signature can serve as a definitive biomarker of microbe-induced oncogenesis.

### Cytotoxic necrotizing factor 1 and tumor progression

Among other toxins secreted by UPEC (Naskar et al., 2023), cytotoxic necrotizing factor 1 (CNF1) has recently been implicated in bladder cancer progression. In bladder cancer cells, research has shown that CNF1 was reported to activate the Ras homolog family member C (RhoC) pathway, which in turn upregulates HIF1α and VEGF expression, thereby driving angiogenesis (Guo et al., 2020). The critical consequence of this cascade is a tumor microenvironment increasingly favorable to cancer growth (Guo et al., 2020). This research identified a novel molecular mechanism by which UPEC infection may contribute to bladder cancer progression: CNF1 → RhoC → HSF1/HSP90α → HIF1α → VEGF → angiogenesis and tumor promotion. Such a pathway not only strengthens the biological plausibility of infection-driven tumorigenesis but also highlights RhoC, HSP90α, HIF1α, and VEGF as potential therapeutic targets. Yet, despite these mechanistic insights, direct evidence that CNF1 plays a causal role in bladder cancer initiation or metastasis remains limited. Further studies will be required to determine whether this bacterial toxin represents merely a contributing factor or a direct driver of bladder oncogenesis.

### ROS-mediated tumorigenesis

*Enterococcus faecalis* is well recognized as a commensal organism of the gut, but it is also a known cause of urinary tract infections. Multiple metagenomic studies have reported its enrichment in bladder cancer tissue, and in one analysis, *Bacteroides* and *Enterococcus* were among the most abundant taxa detected in bladder tumors (Parra-Grande et al., 2022). Clinical associations have also been observed, with certain patients exhibiting improved responses to chemotherapy agents, such as gemcitabine, in the presence of *Enterococcus* (Ginwala et al., 2025). Mechanistic clues come from its capacity to secrete ROS, including superoxide (O₂^⁻^), hydrogen peroxide (H₂O₂), and hydroxyl radicals (Huycke et al., 2002). In colorectal cancer models, ROS generated by *E. faecalis* have been shown to damage epithelial DNA, induce inflammation, and promote chromosomal instability, contributing to adenoma and carcinoma development (Huycke et al., 2002). In addition, collagenolytic strains of *E. faecalis* possess the ability to degrade collagen, a property linked to enhanced metastatic potential in cancer cells (Williamson et al., 2022). *Enterococcus faecalis* may contribute to bladder carcinogenesis through the same mechanisms, promoting DNA damage, chromosomal instability, and chronic inflammation via ROS production and facilitating tissue invasion through collagen degradation. Yet, there is currently no direct experimental demonstration of these mechanisms in bladder cancer.

### Microbial enzymes and ECM remodeling in bladder cancer

The ECM, together with the urobiome, is increasingly recognized as a microenvironmental factor that may contribute to bladder cancer initiation and progression. Interactions between ECM components and bacterial products normally participate in the maintenance of tissue homeostasis. When imbalanced, however, these interactions can generate a protumorigenic niche or increase the likelihood of tumor recurrence (Alfano et al., 2016). Enzymes secreted by bacteria can play a particularly important role in these imbalanced processes. By degrading or remodeling the ECM, they promote tissue injury and microenvironmental disruption, which in turn drive chronic inflammation—a condition long associated with cancer initiation and progression.

The inflammatory milieu facilitates cancer cell invasion, tissue destruction, and remodeling of the tumor niche. Specific examples illustrate this principle. Collagenases degrade collagen, restructuring the ECM and permitting tissue infiltration, with *Pseudomonas* and *Serratia* shown to overexpress these enzymes in bladder cancer patients (Alfano et al., 2016). Elastases, known again to be produced by *Pseudomonas* and *Serratia*, degrade elastin, disrupting tight junctions and inducing inflammation. Hyaluronidases, secreted by *Staphylococcus* and *Streptococcus*, degrade hyaluronic acid, weakening the ECM and promoting tumor cell motility and invasion. Serine proteases, reported in *Aeromonas*, break down multiple ECM proteins, while alkaline proteases, elastases, and phospholipase C, produced by *Staphylococcus aureus*, disrupt ECM structure and destroy cell–cell junctions, damaging bladder tissue integrity (Odunitan et al., 2024; Wu et al., 2018). A striking example comes from large-scale bladder cancer microbiome studies that identified *Stenotrophomonas maltophilia* as being highly enriched within tumor tissue, accounting for up to 61% of sequences in some cases (Li et al., 2025). This opportunistic bacterium is known to secrete multiple extracellular proteases—including serine proteases StmPr1, StmPr2, and StmPr3—as well as lytic enzymes and siderophores, and it possesses diverse metabolic capacities for amino acids, fatty acids, and carbohydrates. The StmPr proteases can degrade host ECM proteins, such as collagen, fibronectin, and fibrinogen, as well as adhesion molecules, tight junctions, and integrins, thereby weakening cell–cell interactions and inducing epithelial detachment (DuMont and Cianciotto, 2017).

Collectively, these findings suggest that microbial enzymes and metabolites—particularly proteases—have the capacity to reshape the bladder tumor microenvironment through ECM degradation, tissue damage, and inflammation induction. Yet, despite these mechanistic insights, definitive causal links between such microbial products and bladder carcinogenesis remain to be established, underscoring the need for targeted experimental studies.

### *Corynebacterium* and nitrogen metabolism in bladder cancer

Multiple independent studies have reported a significant enrichment of *Corynebacterium* species in bladder cancer patients. This genus is notable for its strong urease activity. Urease converts urea into ammonia and generates diverse nitrogen-containing metabolites, such as nitrates, nitrites, and polyamines. Through these reactions, *Corynebacterium* alters urinary pH and fosters the accumulation of alkaline metabolites like ammonia, reshaping the tumor microenvironment to favor cancer cell survival and growth (Sheng et al., 2025a; Soriano and Tauch, 2008). However, the impact of these metabolic activities extends beyond simply changing the biochemical environment. By sustaining chronic, low-grade local inflammation and enhancing the production of immune-modulatory factors, *Corynebacterium* creates a milieu facilitating tumor initiation, early growth, and progression. The activation of nitrogen metabolism pathways, in concert with the increased secretion of inflammatory cytokines, suggests that these bacteria may actively promote tumorigenesis within the bladder. Recent large-scale multi-omics analyses strengthen this view. Distinct microbial community compositional archetypes—so-called “urinetypes”—associated with bladder cancer have been identified, with *Corynebacterium* and *Prevotella* emerging as the dominant taxa characterizing them. These urinetypes occur with striking frequency in bladder cancer patients and are particularly prominent in high-risk subtypes (Sheng et al., 2025a). Together, these findings indicate that microbial shifts are not merely bystander phenomena. Instead, the nitrogen-metabolizing activity of *Corynebacterium* and its partnership with *Prevotella* may contribute directly to the variability seen in bladder cancer progression and, thus, its prognosis of bladder cancer. Such patterns suggest that these taxa could serve as ecological biomarkers for disease classification and prognostic prediction.

### Acetaldehyde-driven genotoxicity in bladder cancer

Microbial metabolism functions as a critical mediator in shaping the chronic inflammatory microenvironment of the bladder. Among its products, genotoxic metabolites, such as acetaldehyde, have been repeatedly implicated in tissue injury and carcinogenic processes. When microbial dysbiosis permits the translocation of bacteria into host tissues, inflammatory cascades are triggered, activating pathways directly tied to tumorigenesis—including Toll-like receptor signaling and the JAK–STAT3, NF-κB, and PI3K–Akt–mTOR pathways. In this setting, microbial products like acetaldehyde and nitrosamines, together with ROS, reactive nitrogen species, and hydrogen sulfide (H₂S), contribute to DNA damage and malignant transformation (Fankhauser and Mostafid, 2018).

In bladder cancer specifically, acetaldehyde has emerged as a compound of particular concern. It can be generated both by the host cell’s metabolism and by microbial activity, and when it accumulates, it is believed to exert genotoxic and tumor-promoting effects. Recent studies have identified *Candida glabrata* as a clinically relevant fungal species in the urinary tract capable of fermenting glucose into acetaldehyde. Persistent exposure of urothelial cells to this metabolite may exacerbate DNA damage, intensifying carcinogenic risk (Nieminen et al., 2009). Epidemiologic findings further reinforce the role of acetaldehyde exposure in contributing to cancer development. While overall associations between alcohol intake and bladder cancer risk appear modest, dose–response analyses reveal that in men and in populations consuming high-proof spirits, incremental increases in alcohol intake (approximately 12 g per drink) are linked to an 8–9% rise in risk (Lao et al., 2021). These findings suggest that cumulative acetaldehyde exposure, rather than alcohol consumption per se, may represent a more relevant determinant of bladder cancer susceptibility. Microbial metabolism contributes to bladder carcinogenesis not merely as a background process but as an active driver of genotoxic stress, chronic inflammation, and metabolic disruption. Acetaldehyde, produced by both bacteria and fungi, is implicated in modulating the urothelial microenvironment. The degree of bladder cancer risk attributable to acetaldehyde-related pathways likely depends on exposure dose, host metabolic capacity, and the composition of the urobiome.

### Hydrogen sulfide’s role in tumor microenvironment remodeling

Recent studies have highlighted H₂S, a microbial metabolite, as an important factor in shaping the tumor microenvironment and promoting the progression of cancers, including bladder cancer. Of particular interest is *Fusobacterium nucleatum*, which has been found to be enriched in both the urine and tumor tissue of bladder cancer patients, distinguishing it as a noteworthy member of the urobiome (Bučević Popović et al., 2018). This organism metabolizes sulfur-containing amino acids, such as methionine and cysteine, through specific enzymatic pathways, making it a major microbial source of H₂S (Wang et al., 2023). At pathological concentrations, H₂S exerts multiple pro-tumorigenic effects. It activates several signaling pathways, such as the autophagy, NF-κB, and MAPK pathways, while simultaneously enhancing inflammatory responses. Experimental models of colorectal, liver, and lung cancers have shown that H₂S promotes the increased secretion of inflammatory cytokines like IL-6 and IL-8, enhances tumor cell survival and motility, and stimulates autophagic pathways—all changes that facilitate the growth of malignant cells.

Evidence from human bladder cancer cohorts reinforces these mechanistic insights. An analysis of the urobiomes of bladder cancer patients revealed significantly higher abundances of *Fusobacterium*, among other taxa, in patients than in healthy individuals (Bučević Popović et al., 2018). Moreover, genera such as *Streptococcus*, *Corynebacterium*, and *Fusobacterium* showed progressive increases as tumor stages progressed, underscoring their possible roles in disease advancement. These shifts suggest that alterations in microbial composition directly contribute to bladder tumor microenvironment remodeling, particularly through the increased production of metabolites such as H₂S. Thus, the pro-tumorigenic effects of H₂S extend beyond its identity as a metabolic byproduct. By driving immune modulation, activating inflammatory and autophagic signaling, and enhancing tumor cell survival and invasiveness, H₂S has emerged as a pivotal mediator linking microbial dysbiosis to bladder cancer progression. There is a clear need for deeper exploration into the tripartite interaction among microbes, their metabolites, and host inflammatory and tumorigenic pathways (Bučević Popović et al., 2018; Wang et al., 2023).

### *Sphingomonas* and sphingolipid metabolism in bladder cancer

Recent studies have repeatedly shown that bacteria of the genus *Sphingomonas* are markedly more abundant in bladder cancer tissue than in non-malignant tissue (Liu et al., 2019; Sheng et al., 2025b). This pattern suggests it is more than just a simple shift in microbial composition, pointing instead to metabolic and functional changes within the tumor microenvironment. A defining feature of *Sphingomonas* is its specialized sphingolipid metabolism. It possesses diverse pathways that generate or transform distinctive metabolites, such as phytosphingosine and sphinganine—lipid mediators that regulate cell proliferation, differentiation, apoptosis, inflammatory responses, immune signaling, and angiogenesis. The dysregulation of sphingolipid metabolism has repeatedly been reported in bladder cancer and other malignancies, and metabolomic analyses directly support a link between the two. In a large-scale study, levels of phytosphingosine and sphinganine were found to be significantly elevated in the urine and bladder tissues of bladder cancer patients (Mlynarczyk et al., 2024; Ponnusamy et al., 2012; Wittmann et al., 2014). These increases were not merely incidental; they correlated with tumor-promoting processes, including cell proliferation, membrane remodeling, microenvironment modulation, immune and inflammatory regulation, and neovascularization. Importantly, sphingoid bases, including phytosphingosine and sphinganine, were more prominently elevated in high-grade than in low-grade bladder cancers, suggesting their association with disease progression and prognosis (Mlynarczyk et al., 2024; Ponnusamy et al., 2012; Wittmann et al., 2014). These findings suggest that *Sphingomonas* enrichment is closely tied to elevated sphingolipid metabolite levels in the bladder, and that these metabolites actively shape the tumor microenvironment and cancer cell physiology. This convergence of microbial ecology and metabolomics provides compelling evidence that sphingolipid pathways—and the microbes that drive them—represent promising biomarkers for the diagnosis and prognosis of bladder cancer and promising therapeutic treatment targets.

### Tryptophan metabolites in bladder cancer

Recent investigations have demonstrated that urinary metabolomic profiling and biomarker discovery represent promising strategies for the diagnosis of bladder cancer. Yet the interpretation of such studies requires caution. A central limitation lies in the difficulty of distinguishing between metabolites originating from host or microbial processes, a distinction that relates directly to both diagnostic accuracy and physiological implications. In a study comparing urinary metabolomes from 100 bladder cancer patients and 100 healthy individuals, several important alterations were identified. Levels of indoleacetic acid (IAA), a tryptophan-derived metabolite, were decreased in bladder cancer patients—a finding that contrasts with the IAA increases observed in cervical, colorectal, and breast cancers (Nizioł et al., 2023). In contrast, oleamide, a lipid metabolite with high discriminatory power, was significantly enriched. Oleamide interacts with cannabinoid receptors CB1 and CB2 to regulate cell growth and motility, and in bladder cancer T24 cells, it has been shown to increase intracellular calcium concentrations. Elevated oleamide levels have also been reported in kidney, laryngeal, and colorectal cancers. Another metabolite, N-α-acetyllysine, was increased in bladder cancer patients’ urine but decreased in prostate cancer patients’ urine, reflecting tumor-specific patterns of lysine derivative metabolism (Nizioł et al., 2023).

Several of the most informative metabolites—including indolelactic acid, IAA, and hippuric acid—have been closely tied to gut microbial metabolism (Su et al., 2022), raising the strong possibility that metabolite shifts in bladder cancer reflect underlying alterations in the gut microbiome. At the same time, mechanistic complexity must be considered: the excessive accumulation of tryptophan metabolites can activate the aryl hydrocarbon receptor and suppress antitumor immunity, thereby enhancing tumor cell survival and proliferation (Opitz et al., 2011). Thus, while urinary metabolite signatures offer powerful clues to the pathophysiology of bladder cancer, their clinical interpretation remains constrained by a critical uncertainty as to whether metabolites derive from the host, from the bladder microbiota, or from the gut microbiota, appearing secondarily in urine. Progress in this area will depend on the development of integrated analyses capable of tracing metabolite origins, coupled with targeted quantification and external validation. Only then can urinary metabolomics establish itself as a clinically reliable, noninvasive biomarker platform for bladder cancer.

### Succinate accumulation in bladder cancer

Recent studies have identified *Porphyromonas somerae* as one of the most strikingly enriched taxa in the urine of patients with MIBC (Nardelli et al., 2024; Russo et al., 2024). This microbial shift appears to represent a bladder cancer-specific urobiome signature, as neither prostate cancer patients nor healthy controls demonstrated a similar shift (Nardelli et al., 2024). The genus *Porphyromonas* comprises Gram-negative, strictly anaerobic, non-motile, non-spore-forming bacteria that have been linked to diverse human diseases. Its best-known member, *P. gingivalis*, is a principal pathogen in periodontal disease (Su et al., 2020). Although the precise contribution of *P. somerae* metabolites to bladder carcinogenesis remains undefined, mechanistic insights can be drawn from related cancers. In endometrial cancer, *P. somerae* has been shown to invade host cells and generate succinate, a metabolite that accumulates intracellularly, where it activates HIF-1α, triggers chronic inflammation, promotes ECM remodeling, and increases oxidative stress. Together, these processes reshape the tumor microenvironment in ways that favor malignant transformation (Crooks et al., 2021; King et al., 2006; Multinu et al., 2020). A similar pathogenic mechanism may operate in bladder cancer. The intracellular invasion of *P. somerae* and subsequent succinate accumulation may contribute to persistent inflammation, ECM remodeling, and reactive oxygen species generation, supporting tumor initiation and progression. However, these associations in bladder cancer remain speculative and are primarily extrapolated from studies in endometrial cancer; further work is needed to establish direct causality. What remains to be clarified is the extent to which these microenvironmental changes, specifically, shape the development and evolution of bladder cancer.

### Microcystin and urothelial carcinogenesis

*Cyanobacteria* are well known for their ability to produce the toxin microcystin, which has been implicated in hepatocellular carcinoma and reported to enhance both the motility and invasiveness of colorectal cancer cells (Svirčev et al., 2010). In an unusual case, a study analyzing microbial communities from bladder cancer patients identified *Cyanobacteria* not only in urine but also within tumor tissue, at relative abundances of 7% and 8%, respectively (Mansour et al., 2020). Given that *Cyanobacteria* are not typically regarded as common members of the urinary microbiome, their detection in both urine and tumor tissue suggests that extrinsic factors can contribute to their presence. Because alterations in the gut microbiome can influence the urobiome, these observations raise the possibility that regional or dietary factors may affect *Cyanobacteria*l prevalence in the bladder (Zhou et al., 2002). Until recently, *Cyanobacteria* had rarely been detected in human urinary samples; their emergence in studies of bladder cancer and other urologic malignancies therefore represents a novel discovery. The detection of *Cyanobacteria* in bladder cancer patients is intriguing (Buratti et al., 2011), but their contribution remains hypothetical. Evidence for microcystin-mediated effects largely comes from liver and colorectal cancer models; in bladder cancer, this link is suggestive rather than established. Clarifying these associations will require follow-up investigations comparing the presence and abundance of microcystin-producing *Cyanobacteria* between bladder cancer patients and healthy controls.

### Biofilms as microenvironmental drivers of bladder cancer

Biofilms cannot be regarded as mere bacterial byproducts; they represent complex communities in which microbes embed themselves within polysaccharide–protein matrices for stable attachment and growth. The first clinical evidence of bacterial biofilms in bladder cancer tissue was reported in a case study of urothelial carcinoma at stage pT2, where coccoid bacterial aggregates were detected within the submucosal layer (Nadler et al., 2021). Such structures create a persistent microbial reservoir capable of driving chronic inflammation and sustained immune activation within the bladder microenvironment. Among taxa repeatedly linked to bladder cancer, *Acinetobacter* has drawn particular attention. Multiple studies have reported its enrichment in urine (Bukavina et al., 2023) and in tumor tissue itself (Liu et al., 2019). Known as a major pathogen in respiratory and urinary infections, *Acinetobacter* species possess strong biofilm-forming capacities and marked abilities to adhere to and invade epithelial cells (McConnell et al., 2013). These features enable the bacterium to degrade mucosal phospholipids, promote bacterial spread, and evade immune surveillance. By sustaining a chronic inflammatory state, such activities may generate a tumor-permissive microenvironment. Notably, *Acinetobacter baumannii* has been detected at high frequency—up to 75.96%—in high-grade urothelial carcinoma patients, suggesting an association with aggressive bladder cancer phenotypes (Murphy and Frick, 2013; Sheng et al., 2025a).

The pathological impact of *Acinetobacter* extends beyond biofilm formation. By disrupting epithelial barriers, altering immune responses, and remodeling the ECM, this genus may amplify existing pro-tumor effects of the urobiome. The ECM itself plays a critical role in cancer initiation, progression, and metastasis (Huang et al., 2021), and interactions between microbial activity and ECM remodeling can promote persistent inflammation. In colon cancer, for example, tissues harboring biofilms displayed markedly elevated levels of the polyamine metabolite N(1),N(12)-diacetylspermine, even those of nonmalignant mucosa tissue (Johnson et al., 2015). These findings show that biofilms not only anchor microbial communities but also amplify metabolic and inflammatory processes within the tumor microenvironment. In bladder cancer, similar dynamics appear to be at work. Biofilm formation has been linked to ECM remodeling, sustained inflammation, and enhanced cellular motility, processes that collectively accelerate tumor invasiveness and recurrence (Huang et al., 2021; Pathoor et al., 2024). Thus, biofilms must be considered to be more than structural curiosities; they constitute active microenvironmental drivers of bladder cancer progression. Their central role in these processes highlights the importance of developing therapeutic strategies that specifically target biofilm formation and maintenance as a means of disrupting tumor-promoting niches.

## The Role of the Microbiota–Metabolite–Immunity Axis in Bladder Cancer and Precision Therapeutic Strategies

For decades, variability in chemotherapy responses and resistance was understood largely in terms of tumor-intrinsic factors, such as genetic mutations or altered drug transporter expression. More recently, however, attention has shifted to the patient’s microbiome and the metabolites it produces, which can profoundly influence anticancer therapies. The inactivation of gemcitabine by microbial enzymes was first demonstrated in pancreatic cancer, where intratumoral *Gammaproteobacteria* converted the drug into inactive derivatives via cytidine deaminase (Geller et al., 2017). Although this evidence originates outside the urinary tract, *Gammaproteobacteria* are also common members of the urinary microbiome. This raises the possibility that a similar mechanism could reduce gemcitabine efficacy in bladder cancer, highlighting the need for targeted studies in this context. Such findings demonstrate that the microbiome is not merely a bystander but a regulator of therapeutic response.

At the same time, other microbial metabolites exert direct anticancer effects. Short-chain fatty acids (SCFAs), particularly those produced by probiotic genera like *Bifidobacterium* and *Lactobacillus*, have emerged as functional molecules with therapeutic potential. Butyrate, for example, has been shown in bladder cancer cell models to induce ROS, activate autophagy, suppress proliferation, and modulate signaling through the miR-139-5p/Bmi-1 axis and AMPK/mTOR pathways (Wang et al., 2020). These results suggest that SCFAs act not as passive byproducts but as active signaling mediators capable of enhancing anticancer treatment.

The therapeutic implications of positive microbiota–metabolite–immunity axis processes are rapidly being advanced through synthetic biology. CRISPR-based engineering is being used to design microbes that deliver selective metabolic products directly to tumors, while other approaches seek to modulate microbial metabolite production itself. Strategies under investigation include probiotic supplementation, the intravesical instillation of microbial metabolites, the targeted elimination of microbes responsible for drug inactivation, and the development of novel drugs derived from microbial metabolites. Ultimately, these projects are leading toward precision medicine strategies in which the urinary and/or gut microbiome of individual patients can be profiled to predict or modify therapeutic outcomes. By integrating the microbiome’s dual capacity as modulators of drug metabolism and regulators of immune response, the field is moving toward a new bladder cancer-treatment paradigm of “microbiome–metabolite–immunity-based personalized therapy”—a paradigm that may soon enter clinical reality.

## Conclusion and Future Perspectives

Bladder cancer, as the most common malignancy of the urinary tract, is increasingly recognized as a disease shaped not only by host genetics and environmental exposures but also by the urinary microbiome and its metabolites. The evidence summarized here demonstrates that dysbiosis of the urobiome contributes to bladder carcinogenesis, progression, and therapeutic response through mechanisms that include genotoxic metabolite production, ECM remodeling, chronic inflammation, and modulation of immune pathways. Distinct microbial signatures have been observed across disease stages, between primary and recurrent cases, and even in relation to treatment outcomes, underscoring their potential as diagnostic and prognostic biomarkers. At the same time, specific microbial metabolites such as short-chain fatty acids highlight the therapeutic opportunities that may be harnessed through targeted microbiome modulation. Integrating urinary and gut microbiome profiling with metabolomics and host immunologic signatures will likely accelerate the development of precision oncology approaches in bladder cancer. Moving forward, large multicenter studies, standardized sampling methods, and mechanistic validation in experimental models are essential to confirm causal relationships and to translate these insights into clinical applications. In summary, the microbiota–metabolite–immunity axis offers both mechanistic insights into bladder cancer biology and a promising framework for next-generation diagnostic and therapeutic strategies. We anticipate that advances in this area will soon reshape how bladder cancer is classified, monitored, and treated in clinical practice.

## Figures and Tables

**Table 1.** Summary of studies characterizing the urinary and tissue microbiome in bladder cancer patients

Specimen (Study/Year)	Case/Control	NMIBC/MIBC	Gender	Enriched genera in BC
Midstream urine, Bladder tissue (Bučević Popović et al., 2018)	12/11	Primary NMIBC (10)/Recurrent NMIBC (2)	M	*Fusobacterium*, *Actinobaculum*, *Facklamia*, *Campylobacter*, *Subdoligranulum*
Midstream urine (Wu et al., 2018)	31/18	26/5	M	*Acinetobacter*, *Anaerococcus*, *Rubrobacter*, *Sphingobacterium*, *Atopostipes*, *Geobacillus*, *Herbaspirillum*, *Porphyrobacter*
				*Bacteroides* were particularly enriched in high-risk patients
Bladder tissue (Liu et al., 2019)	22/0	5/17	M	*Cupriavidus* spp., unclassified *Brucellaceae*, *Acinetobacter*, *Escherichia*-*Shigella*, *Sphingomonas*, *Pelomonas*, *Ralstonia*, *Anoxybacillus*, *Geobacillus*
Midstream urine (Zeng et al., 2020)	62/19	51/11	M	Recurrence NMIBC: *Anoxybacillus*, *Micrococcus*, *Staphylococcus*, *Streptococcus*, *Prevotella*, *Corynebacterium*_1, *Oscillobacter*, *Peptoniphilus*, *Bacteroides*
Transurethral resectoscopy urine, Bladder tissue (Mansour et al., 2020)	10/0	6/4	F, M	Urine samples: *Lactobacillus*, *Corynebacterium*, *Streptococcus*, *Staphylococcus*
				Tissue samples: *Bacteroides*, *Akkermansia*, *Klebsiella*, *Enterobacter*, *Clostridium* sensu stricto.
Midstream urine, Tissue (Pederzoli et al., 2020)	49/59	Mixed	F, M	Urine samples: *Klebsiella* (only in female)
				Tissue samples: *Burkholderia* (both in male and female)
Urine (midstream or catheter) (Hussein et al., 2021)	43/10	29/14	F, M	Both type: *Actinomyces*, *Achromobacter*, *Brevibacterium*, *Brucella*
				NMIBC: *Cupriavidus*
				MIBC: *Haemophilus*, *Veillonella*
				BCG responder (NMIBC): *Serratia*, *Brochothrix*, *Negativicoccus*, *Escherichia*-*Shigella*, *Pseudomonas*
Urine (midstream or catheter), bladder washout (Oresta et al., 2021)	51/10	Mixed	F, M	Midstream urines: *Streptococcus*, *Enterococcus*, *Corynebacterium*, *Fusobacterium*
				Bladder washouts: *Burkholderiaceae*
				Catheterized urines: *Veillonella*, *Corynebacterium*
Bladder tissue (Parra-Grande et al., 2022)	32/0	Mixed	F, M	*Bacteroidetes*, *Escherichia*‑*Shigella*, *Enterococcus*, *Barnesiella*, *Parabacteroides*, *Prevotella*, *Alistipes*, *Staphylococcus*
Midstream urine (Chorbińska et al., 2023)	18/7	Mixed	F, M	*Howardellagenus*, *Streptococcus* *anginosus*.
				*Lactobacillus was more frequent in BCG-treated patients.
Bladder tumor tissue (Sun et al., 2023)	22/0	7/15	F, M	Both type: *Ralstonia*, *Cutibacterium*
				NMIBC: *Bacteroides*, *Staphylococcus*, *Acinetobacter*
				MBIC: *Enterococcus*, *Sphingomonas*, *Metamycoplasma*
Catheterized urine (Heidrich et al., 2024)	32/41	32/0	M	No significant differences in microbiota composition (NMIBC vs. controls)
				* Association of *Lactobacillus*, *Streptococcus*, *Cutibacterium* with better BCG response
Midstream urine, bladder tissue (Bilski et al., 2024)	41/0	22/19	F, M	Male: *Campylobacter*, *Sphingobium*, *Haemophilus*, *Aeribacillus*, *Peptococcus*, *Alcaligenes*, *Actinomyces*, *Pseudomonas*, *Acinetobacter*, *Proteus*
				Female: *Salmonella*, *Romboutsia*, *Enterobacter*
Midstream urine (Sheng et al., 2025b)	170/0	Mixed	F, M	Primary BCa: *Sphingomonas*, *Corynebacterium*, *Capnocytophaga*, *Massilia*, *Aquabacterium*
		Primary (39) vs Recurrent (39)		Recurrent BCa: *Aeromonas*, *Cupriavidus*, *Bradyrhizobium*
Midstream urine (Ginwala et al., 2025)	55/13	Mixed	F, M	*Enterobacteriales*, *Flavobacterium*, *Varicubaculum*, *Facklamia*
				Chemotherapy non-responders: *Granulicatella*, *Proteus*
				Chemotherapy responders: *Enterococcus* *faecalis*

**Table 2.** Microbial metabolites/effectors linked to bladder cancer, associated taxa, and mechanistic effects

Metabolite/Effector	Representative microbial taxa	Mechanistic effect in bladder cancer
Colibactin (Chagneau et al., 2021; Dziubańska-Kusibab et al., 2020; Nougayrède et al., 2006)	*Escherichia coli* (pks^+^ strains)	DNA alkylation, double-strand breaks, mutational signature
Cytotoxic Necrotizing Factor 1 (CNF1) (Guo et al., 2020)	Uropathogenic *E. coli*	RhoC–HIF1α–VEGF pathway activation → angiogenesis
ECM-remodeling enzymes (Alfano et al., 2016; DuMont and Cianciotto, 2017; Odunitan et al., 2024)	*Pseudomonas, Serratia, Staphylococcus, Stenotrophomonas *	ECM degradation, junction disruption, enhanced invasion
Succinate (Crooks et al., 2021; Nardelli et al., 2024)	*Porphyromonas *	HIF-1α stabilization, ECM remodeling, ROS generation
Microcystin (Mansour et al., 2020; Svirčev et al., 2010)	*Cyanobacteria*	DNA damage, enhanced invasiveness, environmental influence
Reactive Oxygen Species (ROS) (Huycke et al., 2002; Williamson et al., 2022)	*Enterococcus*	DNA damage, inflammation, epithelial injury
Nitrogen metabolism products (Sheng et al., 2025a; Soriano and Tauch, 2008)	*Corynebacterium*, *Prevotella*	pH shift, nitrogen metabolite buildup, chronic inflammation
Acetaldehyde (Lao et al., 2021; Nieminen et al., 2009)	*Candida*	DNA damage, chronic inflammation
Hydrogen sulfide (H₂S) (Wang et al., 2023)	*Streptococcus,Corynebacterium, Fusobacterium*	NF-κB/MAPK activation, immune modulation, autophagy
Sphingolipid metabolites (Mlynarczyk et al., 2024; Ponnusamy et al., 2012)	*Sphingomonas*	Cell proliferation, angiogenesis, immune modulation
Tryptophan metabolites (Nizioł et al., 2023; Opitz et al., 2011)	Bladder or gut originated microbiota	AHR activation, immunosuppression
Biofilms (Johnson et al., 2015; Nadler et al., 2021)	*Acinetobacter*	ECM remodeling, immune evasion, chronic inflammation
